# Cost/efficacy analysis of preferred Spanish AIDS study group regimens and the dual therapy with LPV/r+3TC for initial ART in HIV infected adults

**DOI:** 10.7448/IAS.17.4.19603

**Published:** 2014-11-02

**Authors:** Josép M Gatell, José R Arribas, Pablo Lázaro, Antonio Blasco

**Affiliations:** 1 Head Infectious Diseases and AIDS Units, Hospital Clinic-IDIBAPS, Barcelona, Spain; 2 HIV Unit, La Paz Hospital. IdiPAZ, Madrid, Spain; 3 Research, Advanced Techniques in Health Services Research (TAISS), Madrid, Spain

## Abstract

**Introduction:**

The National AIDS Plan and the Spanish AIDS study group (GESIDA) panel of experts propose “preferred regimens” of antiretroviral treatment (ART) as initial therapy in HIV-infected patients for 2013 [1]. All these regimens are triple therapy regimens. The Gardel Study assessed the efficacy and safety of a dual therapy (DT) combination of lopinavir/ritonavir (LPV/r) 400/100 mg BID+ lamivudine (3TC) 150 mg BID [2]. The objective of this study is to evaluate the costs and efficiency of initiating treatment with the GESIDA “preferred regimens” and DT.

**Materials and Methods:**

Economic assessment of costs and efficiency (cost/efficacy) through decision tree analysis models. Efficacy was defined as the probability of having viral load <50 copies/mL at week 48, in an intention-to-treat analysis. Cost of initiating treatment with an ART regime was defined as the costs of ART and its consequences (adverse effects, changes of ART regime and drug resistance tests) during the first 48 weeks. The payer perspective (Spanish National Health System) was applied considering only differential direct costs: ART (official prizes), management of adverse effects, resistance tests, and determination of HLA B*5701. The setting is Spain and the costs are those of 2013. A sensitivity deterministic analysis was conducted, building three scenarios for each regime: base, most favourable and most unfavourable cases.

**Results:**

In the base case scenario, the cost of initiating treatment ranges from 5138 euros for DT, to 12,059 euros for tenofovir DF/emtricitabine (TDF/FTC)+raltegravir (RAL). The efficacy ranges between 0.66 for abacavir (ABC)/3TC+LPV/r and ABC/3TC+atazanavir (ATV)/r, and 0.88 for DT. Efficiency, in terms of cost/efficacy, varies between 5817 and 13,930 euros per responder at 48 weeks, for DT and TDF/FTC+RAL respectively. DT is the most efficient regimen in the most favourable (5503 euros per responder) and most unfavourable (6169 euros per responder) scenarios.

**Conclusions:**

Considering the ART official Spanish prizes, the most efficient regimen was DT, followed by the triple therapy with non-nucleoside containing regimens. The sensitivity analysis confirms the robustness of these findings.
